# A simple mathematical model for the evaluation of the long first wave of the COVID-19 pandemic in Brazil

**DOI:** 10.1038/s41598-021-95815-9

**Published:** 2021-08-12

**Authors:** Yuanji Tang, Tamires D. A. Serdan, Amanda L. Alecrim, Diego R. Souza, Bruno R. M. Nacano, Flaviano L. R. Silva, Eliane B. Silva, Sarah O. Poma, Matheus Gennari-Felipe, Patrícia N. Iser-Bem, Laureane N. Masi, Sherry Tang, Adriana C. Levada-Pires, Elaine Hatanaka, Maria F. Cury-Boaventura, Fernanda T. Borges, Tania C. Pithon-Curi, Marli C. Curpertino, Jarlei Fiamoncini, Carol Gois Leandro, Renata Gorjao, Rui Curi, Sandro Massao Hirabara

**Affiliations:** 1 Applied NanoFemto Technologies, LLC, Lowell, MA USA; 2 Interdisciplinary Program of Health Sciences, Cruzeiro do Sul University, Rua Galvao Bueno, 868, Liberdade, Sao Paulo, SP 01506-000 Brazil; 3 Kaiser Southern California Permanente Medical Group, Riverside, CA 92505 USA; 4 Butantan Institute, Sao Paulo, Brazil; 5 Medical School, Faculdade Dinâmica do Vale do Piranga, Ponte Nova, MG Brazil; 6 Laboratory of Epidemiological and Computational Methods in Health, Department of Medicine and Nursing, Universidade Federal de Viçosa, Viçosa, MG Brazil; 7 School of Pharmaceutical Sciences, University of Sao Paulo, Sao Paulo, Brazil; 8 Food Research Center (FoRC), Sao Paulo, Brazil; 9 Federal University of Pernambuco, Recife, Brazil

**Keywords:** Epidemiology, Viral infection, Computational models

## Abstract

We propose herein a mathematical model to predict the COVID-19 evolution and evaluate the impact of governmental decisions on this evolution, attempting to explain the long duration of the pandemic in the 26 Brazilian states and their capitals well as in the Federative Unit. The prediction was performed based on the growth rate of new cases in a stable period, and the graphics plotted with the significant governmental decisions to evaluate the impact on the epidemic curve in each Brazilian state and city. Analysis of the predicted new cases was correlated with the total number of hospitalizations and deaths related to COVID-19. Because Brazil is a vast country, with high heterogeneity and complexity of the regional/local characteristics and governmental authorities among Brazilian states and cities, we individually predicted the epidemic curve based on a specific stable period with reduced or minimal interference on the growth rate of new cases. We found good accuracy, mainly in a short period (weeks). The most critical governmental decisions had a significant temporal impact on pandemic curve growth. A good relationship was found between the predicted number of new cases and the total number of inpatients and deaths related to COVID-19. In summary, we demonstrated that interventional and preventive measures directly and significantly impact the COVID-19 pandemic using a simple mathematical model. This model can easily be applied, helping, and directing health and governmental authorities to make further decisions to combat the pandemic.

## Introduction

The combat of the COVID-19 pandemic imposes various challenges and requires extensive efforts at several levels to implement and follow control measures for preventing the fast dissemination of the severe acute respiratory syndrome coronavirus 2 (SARS-CoV-2)^1–4^. Scientists worldwide are testing numerous drugs to treat COVID-19 and developing vaccines against the SARS-CoV-2^5–8^. Some countries already started their vaccination programs against COVID-19, but the velocity of immunization still is shallow, with limited vaccine disposal. Thus, until this moment, well-established and stringent social distancing/isolating and preventive measures continue to be essential to reduce the rapid SARS-CoV-2 dissemination^2,9,10^. In addition, knowledge of the behavior and transmission of SARS-CoV-2 predicted via computer technology and mathematical models is essential in planning strategies to control the pandemic^11^. In particular, measures are required to prevent overloading health care systems and overutilizing limited medical resources in treating COVID-19 patients^3^.

Researchers have developed several mathematical models for epidemiological predictions of the SARS-CoV-2 dissemination^12–15^, most of them based on the classical model SIR (Susceptible–Infected–Recovered), initially described by Kermack and McKendrick^16^. However, because this pandemic is unprecedented with multiple influencing factors, the mathematical models developed are not completely accurate and still need validation^3,13^. In a comparative study, Anirudh^3^ evaluated the accuracy of several mathematical models used for the COVID-19 pandemic prediction. Most of them are based on the SIR and its derivations, including the Susceptible–Exposed–Infectious–Recovered (SEIR)^17^, Susceptible–Exposed–Infectious–Recovered–Undetectable (SEIRU)^18^, Simple Susceptible Infected–Recovered–Deaths (SIRD)^19^, Susceptible–Exposed–Infective–Recovered–Quarantine (SEIRQ)^20^, Auto-regressive Integrated Moving Average (ARIMA)^21^, Susceptible–Infected–Diagnosed–Ailing–Recognized–Threatened–Healed–Extinct (SIDARTHE)^22^, among other models. All evaluated models presented differences higher than 10% between the predicted and the real number of cases and only two models, ARIMA and SEIRQ, had a difference lower than 20%^3^.

Nowadays, Brazil is the third country in the number of infected people and the second in fatalities. Seven months after the first case (September 2020), the COVID-19 pandemic in Brazil was still alarming. Compared to other countries (e.g., European and Asian countries), Brazil’s epidemic remained for a longer time, which can be related to the specific regional geographical characteristics, population density, socioeconomic inequality, access to the health care system, local and State government management of the disease, and people’s awareness^23–25^. However, mathematical models to estimate and predict the pandemic characteristics and dissemination features in different Brazilian states and cities are still lacking.

We analyzed all Brazilian states and their respective capitals in this work, considering various local interfering factors in each state and city. In addition, we tested our mathematical model’s precision by analyzing specific periods with few or minimal interferences and how the governmental management influenced the epidemiological curve. Our findings are important for understanding the COVID-19 disease spreading dynamics, guiding the Federal, State, and Municipal governments to make fundamental decisions, and plan Public Healthcare programs for controlling the pandemic.

## Methods

We collected all data and information of this work from publicly available online sources, including the Brazilian Ministry of Health (https://covid.saude.gov.br/), Municipal and State Health Secretaries, and WHO (https://covid19.who.int/) websites. We used the number of cumulative cases of COVID-19 from February 26, 2020, to September 30, 2020. We obtained Specific information about the quarantine, lockdown, reopening, school class returning, and flexibilization from the websites of the Municipal and State Health Secretaries. In addition, we performed the analysis of Brazil, its 26 states and their capitals, and the Federative Unit. Brazilian states and the Federative Unit are located in five regions: (1) Southeast: Sao Paulo (SP), Rio de Janeiro (RJ), Minas Gerais (MG), and Espirito Santo (ES); (2) South: Parana (PR), Santa Catarina (SC), and Rio Grande do Sul (RS); (3) Midwest: Goias (GO), Mato Grosso (MT), Mato Grosso do Sul (MS), and the Federative Unit (Distrito Federal—DF); (4) North: Acre (AC), Amazonas (AM), Amapa (AP), Para (PA), Rondonia (RO), Roraima (RR), and Tocantins (TO); and (5) Northeast: Alagoas (AL), Bahia (BA), Ceara (CE), Maranhao (MA), Paraiba (PB), Pernambuco (PE), Piaui (PI), Rio Grande do Norte (RN), and Sergipe (SE).

### Growth rate model (GRM)

We used a simplified mathematical model to predict the COVID-19 pandemic in different Brazilian states and their capitals, based on the growth rate model (GRM), according to Tang and Shang’s model^26^ with some modifications. Briefly, growth rate (μ) was calculated by following equation:

1 $$ \mu \left( {\text{t}} \right) = \alpha {\text{t}} \times \exp \left( {\beta {\text{t}}} \right) $$

where α and β are, respectively, growth factor and decay factor at a specific time (t). The α factor is associated with human behaviors, including population density, medical condition, government policy, society environment, and public health service. The β factor is related to the nature factor, including the Sars-CoV-2 spreading dynamics in a specific local or regional.

The GRM requires the cumulative total cases of infected people over time. We used the following steps for the prediction of the COVID-19 pandemic in different Brazilian states and cities.

1. Calculation of the growth rate by the equation:
  2 $$ {\text{m}} = \left( {{\text{Nt}}{-}{\text{Nt}} - {1}} \right)/{\text{Nt}} - {1}; $$
2. Calculation of the 7-days average of the growth rate to minimize the variation among different days of the week and delay notifications, especially during the weekends;
3. Determination of the a and b factors by exponentially plotting the points (t × growth rate) in a stable period (~ 60 days, with an *r*2 > 0.7);
4. Calculation of the growth rate by using the equation: m = αt × exp βt,
5. Estimation of the Nt using Eq. (3):
  3 $$ {\text{Nt}} = \left( {{1} + {\text{mt}}} \right) \times {\text{Nt}} - {1}. $$

We plotted the graphics with the significant governmental decisions about the COVID-19 pandemic combat to evaluate the temporal association between these decisions and the growth of the disease dissemination.

### Relationship between the growth rate model and the hospitalized patients

Analysis of the predicted number of new cases of COVID by the growth rate model and the total number of symptomatic patients admitted in hospitals, as well as the number of deaths related to COVID-19, was performed for Brazil and the five Brazilian regions (Southeast, South, Midwest, North, and Northeast) from the 8th (Feb 16–22) to the 44th (October 25–31) epidemiological week of 2020.

## Results

The high heterogeneity and complexity of the regional/local characteristics and governmental authorities among Brazilian states and cities directly influenced the COVID-19 spreading dynamics. As a result, there were different disease epidemic curves. Our model based on a stable period for each Brazilian state and city, with reduced or minimal interference on new cases growth rate, predicts the COVID-19 epidemics in the different states and cities. Thus, we attenuated the interference of external factors on the epidemics curve, and the prediction presented an excellent accuracy in most Brazilian states and cities, mainly in short periods (few weeks). Initially, we selected ~ 60 days for the analysis, but some Brazilian states and cities required shorter or longer periods for the study (average, the period used was 74.8 ± 2.8 days). The value of *r*2 was higher than 0.7 for all Brazilian states and capitals, except Porto Alegre (*r*2 = 0.68) and Florianopolis (*r*2 = 0.57). For the whole of Brazil, we used 64 days, with *r*2 = 0.95.

We found out that our predictions are more accurate in short periods than in long periods (Table 1). For the whole Brazil, we found low discrepancy between the predicted and the reported cumulative number of cases until 28 days (< 10%). At day 60, the difference increased to + 22.3% and at day 90 to + 30.5%. For the 26 Brazilian states and the Federative Unit, at day 7, two states presented difference higher than 10%: SC (+ 18.5%) and MT (− 15.3%); at day 14, six states: SC (+ 18%), MT (− 14.6%), MS (+ 11.7%), GO (+ 11.2), TO (+ 15.4%), and AL (+ 11.1%); at day 21, 10 states: RS (+ 12.6%), SC (+ 17.7%), MT (− 19.3%), MS (+ 14.1%), GO (+ 15.6%), DF (+ 10.2%), AM (+ 12.8%), TO (25.2%), PB (+ 11.1%), and PE (+ 10.6%); at day 28, 13 states: RJ (+ 12.6%), ES (− 12.9%), PR (+ 12.6%), RS (+ 16.3%), SC (+ 17.3%), MT (− 22.8%), MS (+ 18.1%), DF (+ 13.2%), AM (+ 16.3%), TO (+ 33%), AL (+ 19.2%), PB (+ 13.9%), and PE (+ 15.9%). Even after 28 days, the discrepancy was higher than 20% only in two states: MT and TO. At day 60, six of 22 states with prediction presented low variance between predicted and reported cumulative number of cases: BA (+ 7.9%), CE (− 1.4%), MA (+ 7.4%), PI (+ 1.1%), RN (+ 1.2%), and SE (− 2.4%). At day 90, one of seven states with prediction showed low variance between predicted and reported number: CE (+ 1.1%).

**Table 1:** Predictions for the whole Brazil and its states using the growth rate model for new cases.

State	Period for the calculation	Exponential regression (r2)	% variation between predicted versus reported number of cumulative cases								
			7 days	14 days	2 days	28 days	45 days	60 days	75 days	90 days	105 days
BRA	04.25–06.27	0.952	+ 2.4	+ 4.2	+ 4.7	+ 9.0	+ 16.4	+ 22.3	+ 26.7	+ 30.5	+ 31.4 (95 days)
SP	04.08–06.30	0.875	+ 2.2	+ 4.5	+ 2.5	+ 7.0	+ 20.6	+ 23.7	+ 25.5	+ 26.3	+ 26.6 (92 days)
RJ	05.12–07.17	0.940	+ 3.1	+ 4.0	+ 6.2	+ 12.6	+ 21.9	+ 26.1	+ 28.9 (75 days)	–	–
MG	05.23–08.05	0.806	+ 2.1	+ 4.2	+ 6.4	+ 8.5	+ 13.3	+ 16.2 (56 days)	–	–	–
ES	04.19–06.30	0.807	− 0.3	− 3.6	− 8.2	− 12.9	− 18.4	− 24.6	− 29.7	− 31.2	− 30.9 (92 days)
PR	06.19–08.17	0.951	+ 3.0	+ 6.6	+ 9.8	+ 12.6	+ 19.8 (44 days)	–	–	–	–
RS	05.02–08.20	0.800	+ 3.68.3	+ 12.6	+ 16.3	+ 19.4 (41 days)	–	–	–	–	–
SC	06.21–08.27	0.840	+ 18.5	+ 18.0	+ 17.7	+ 17.3	+ 17.1 (34 days)	–	–	–	–
MT	05.22–07.10	0.792	− 15.3	− 14.6	− 19.3	− 22.8	− 41.9	− 57.9	− 63.6	− 65.4 (82 days)	–
MS	05.19–07.20	0.859	+ 7.0	+ 11.7	+ 14.1	+ 18.1	+ 25.6	+ 30.2	+ 32.6 (72 days)	–	–
GO	07.21–09.09	0.927	+ 5.8	+ 11.2	+ 15.6 (21 days)	–	–	–	–	–	–
DF	06.02–07.24	0.940	+ 3.3	+ 6.8	+ 10.2	+ 13.2	+ 19.3	+ 22.0+ 23.5 (68 days)	–	–	–
AC	05.29–07.27	0.941	+ 3.2	+ 5.5	+ 6.9	+ 9.2	+ 13.4	+ 17.7	+ 18.9 (65 days)	–	–
AP	04.23–07.21	0.828	− 0.9	− 0.9	+ 0.6	+ 1.9	+ 9.0	+ 12.3	+ 14.6 (71 days)	–	–
AM	05.03–06.30	0.976	+ 5.3	+ 9.0	+ 12.8	+ 16.3	+ 23.6	+ 28.6	+ 32.3	+ 36.8	+ 38.0 (92 days)
PA	05.12–07.15	0.983	+ 2.0	+ 3.3	+ 5.8	+ 9.3	+ 17.8	+ 22.5	+ 26.7	+ 27.5 (77 days)	–
RO	04.20–07.10	0.861	− 3.0	+ 3.0	+ 4.2	+ 6.5	+ 9.6	+ 12.9	+ 16.2	+ 17.1 (82 days)	–
RR	04.17–08.22	0.801	− 0.8	− 1.2	− 1.7	− 0.2	+ 1.8 (39 days)	–	–	–	–
TO	04.28–06.20	0.971	+ 6.4	+ 15.4	+ 25.2	+ 33.0	+ 54.9	+ 67.8	+ 75.4	+ 78.8	+ 80.4 (102 days)
AL	05.24–07.21	0.984	+ 5.6	+ 11.1	+ 15.7	+ 19.2	+ 23.0	+ 25.2	+ 27.0 (71 days)	–	–
BA	03.26–07.17	0.825	+ 3.2	+ 5.2	+ 6.0	+ 7.7	+ 8.8+ 7.9	+ 5.4 (75 days)	–	–	–
CE	03.24–06.30	0.864	− 1.4	− 3.6	− 5.8	− 4.7	− 1.9	− 1.4	− 0.4	+ 1.1	+ 1.4 (92 days)
MA	04.05–06.30	0.919	+ 0.2	− 0.6	− 2.7	− 2.3	+ 2.2	+ 7.4	+ 11.2	+ 14.6	+ 15.1 (92 days)
PB	05.17–07.19	0.988	+ 4.8	+ 8.7	+ 11.1	+ 13.9	+ 19.0	+ 22.8	+ 25.6 (73 days)	–	–
PE	04.11–06.16	0.968	+ 2.5	+ 5.3	+ 10.6	+ 15.9	+ 30.3	+ 38.9	+ 44.5	+ 48.8	+ 52.0 (105 days)
PI	04.16–07.23	0.939	+ 0.5	+ 0.9	+ 0.7	+ 0.7	+ 0.2	+ 1.1	+ 2.6 (69 days)	–	–
RN	05.13–07.21	0.701	− 0.5	+ 1.0	+ 3.0	+ 0.08	+ 0.6	+ 1.2	+ 2.7 (71 days)	–	–
SE	05.06–07.15	0.705	+ 4.3	+ 8.0	+ 8.2	+ 6.5	+ 2.0	− 2.4	− 5.5	− 5.8 (77 days)	–

Numbers in parentheses indicate the last day of the prediction.

**Figure 1:**
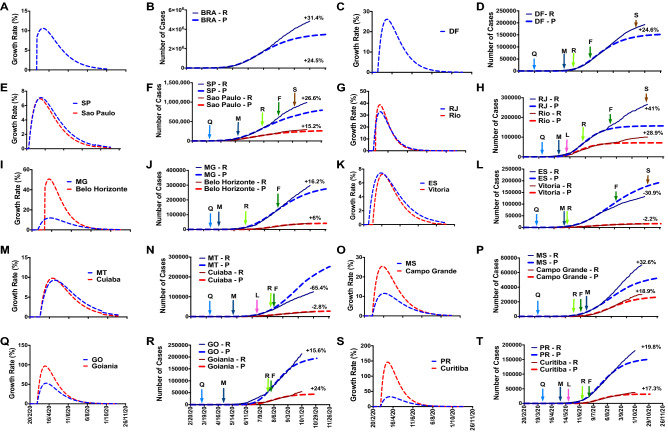
(**A**), (**C**), (**E**), (**G**), (**I**), (**K**), (**M**), (**O**), (**Q**), (**S**): Cumulative COVID-19 cases; and (**B**), (**D**), (**F**), (**H**), (**J**), (**L**), (**N**), (**P**), (**R**), (**T**): Growth rate of COVID-19 in Brazil, Distrito Federal (DF), Sao Paulo (SP), Rio de Janeiro (RJ), Minas Gerais (MG), Espirito Santo (ES), Mato Grosso (MG), Mato Grosso do Sul (MS), Goias, and Parana (PR), respectively, with their respective capitals. − R: reported number; − P: predicted number; Q: quarantine; L: lockdown, M: mask use; R: reopening; F: flexibilization; S: school returning.

**Figure 2:**
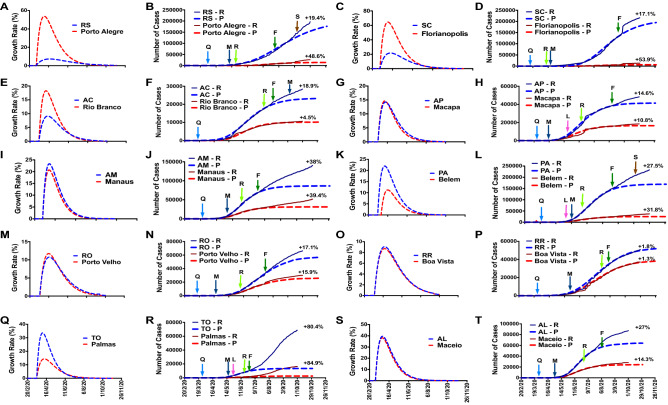
(**A**), (**C**), (**E**), (**G**), (**I**), (**K**), (**M**), (**O**), (**Q**), (**S**): Cumulative COVID-19 cases; and (**B**), (**D**), (**F**), (**H**), (**J**), (**L**), (**N**), (**P**), (**R**), (T): Growth rate of COVID-19 in Rio Grande do Sul (RS), Santa Catarina (SC), Acre (AC), Amapa (AP), Amazonas (AM), Para (PA), Rondonia (RO), Roraima (RR), Tocantins (TO), and Alagoas (AL), respectively, with their respective capitals. − R: reported number; − P: predicted number; Q: quarantine; L: lockdown, M: mask use; R: reopening; F: flexibilization; S: school returning.

**Figure 3:**
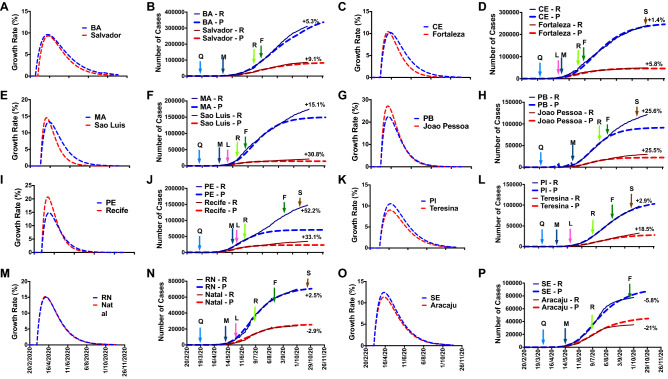
(**A**), (**C**), (**E**), (**G**), (**I**), (**K**), (**M**), (**O**): Cumulative COVID-19 cases; and (**B**), (**D**), (**F**), (**H**), (**J**), (**L**), (**N**), (**P**): Growth rate of COVID-19 in Bahia (BA), Ceara (CE), Maranha (MA), Paraiba (PB), Pernambuco (PE), Piaui (PI), Rio Grande do Norte (RN), and Sergipe (SE), respectively, with their respective capitals. − R: reported number; − P: predicted number; Q: quarantine; L: lockdown, M: mask use; R: reopening; F: flexibilization; S: school returning.

Figures 1, 2, and 3 show the whole country data and 26 Brazilian states and their capitals and the Federative Unit. In general, we observed specific COVID-19 epidemics dynamics among the Brazilian states. We classified the Brazilian states in the following situations: (1) states with expected number of cumulative cases close to the predicted number (registered cases are ± 10% deviation of the predicted number); (2) states with positive number of cumulative cases (registered cases are > 10% deviation of the predicted number); and (3) states with negative number of cumulative cases (registered cases < 10% deviation of the predicted number) on September 30, 2020. It is important to report that at the end of the analyzed period (September 30), the Brazilian states and cities are at different time points after the prediction because they presented different periods of stabilization of the growth rate (Table 1). According to the our classification, six Brazilian states presented an expected number of cumulative cases for COVID-19: BA (+ 5.3%), CE (+ 1.4%), PI (+ 2.9%), RN (+ 2.5%), RR (+ 1.8%), and SE (− 5.8%); two presented negative number: ES (− 30.9%) and MT (− 65.4%); and 19 positive number: AC (+ 18.9%), AL (+ 27%), AM (+ 38%), AP (+ 14.6%), GO (+ 15.6%), MA (+ 15.1%), MG (+ 16.2%), MS (+ 32.6%), PA (+ 17.5%), PB (+ 25.6%), PR (+ 19.8%), PE (52.2%), RJ (+ 41%), RO (+ 17.1%), RS (+ 19.4%), SC (+ 17.1%), SP (+ 26.6%), and TO (+ 80.4%). In addition, DF (+ 23.5%) also had a positive reported number. In general, the capitals of the Brazilian states followed the same tendency of the predicted number, excepting the capitals of five states: AC (+ 18.9% in AC vs + 4.5% in Rio Branco), ES (− 30.9% in ES vs − 2.2% in Vitoria), MG (+ 16.2% in MG vs + 6% in Belo Horizonte), MT (− 65.4% in MT vs − 2.8% in Cuiaba), PI (+ 2.9% in PI vs + 18.5% in Teresina), and SE (− 5.8% in SE vs − 21% in Aracaju).

We reported that the outside factors significantly modified the COVID-19 epidemics. For instance, the Sao Paulo state, the most populous Brazilian state (~ 46 million people), and the epicenter of the COVID-19 pandemic in Brazil, established various governmental preventive and protective measures and public health policies at the beginning of the pandemic (February 2020)^27^. The government decreed quarantine (March 2020) and vs determined mandatory mask use (May 2020). On July 1, 2020, most of the Sao Paulo state started to reopen some trading segments, including shopping, commerce, and services (with restriction to 20% of the maximum capacity and 4 h per day), with more flexibilization and reopening on August 7 (40% of the total and 8 h per day). As we can see in Figs. 1, 2 and 3, the reopening and flexibilization procedures harmed the COVID-19 epidemic curve, with a more significant impact on other cities than the capital (Sao Paulo City). According to our mathematical model, the number of cumulative cases in Sao Paulo state was 26.6% higher than the predicted number on September 30. COVID-19 testing in SP state increased from around 890,000 in June to about 1,380,000 in July (+ 54%), contributing to the reported number elevation.

Other 17 Brazilian states also exhibited a negative impact: AC, AL, AM, AP, GO, MA, MG, MS, PA, PB, PR, PE, RJ, RO, RS, SC, and TO. These findings suggest that the preventive procedures and social distancing/isolating were not efficacious to avoid the SARS-CoV-2 spread. The reopening and flexibilization of the trading segments contributed to the growing COVID-19 epidemics in these states. On the other hand, two Brazilian states (ES and MT) presented a reduction in the cumulative number of cases compared to the predicted number. This observation indicates that successful implementation of outside factors and people were aware of the government preventive measures and social distancing/isolating. In the same direction, six Brazilian states reported a similar number of cumulative cases for COVID-19 to the predicted number: BA, CE, PI, RN, RR, and SE, suggesting that the imposed governmental interventions and people awareness were sufficient in combating the disease.

In five Brazilian states, the capitals presented a higher growth rate than the whole state: MG, GO, RS, AC, and PE. In seven states, the growth rate was similar between the capital and the entire state: SP, MT, AM, RO, BA, MA, and RN. In three states, the growth rate was lower in respective capitals than in PI, SE, and TO whole states.

**Figure 4:**
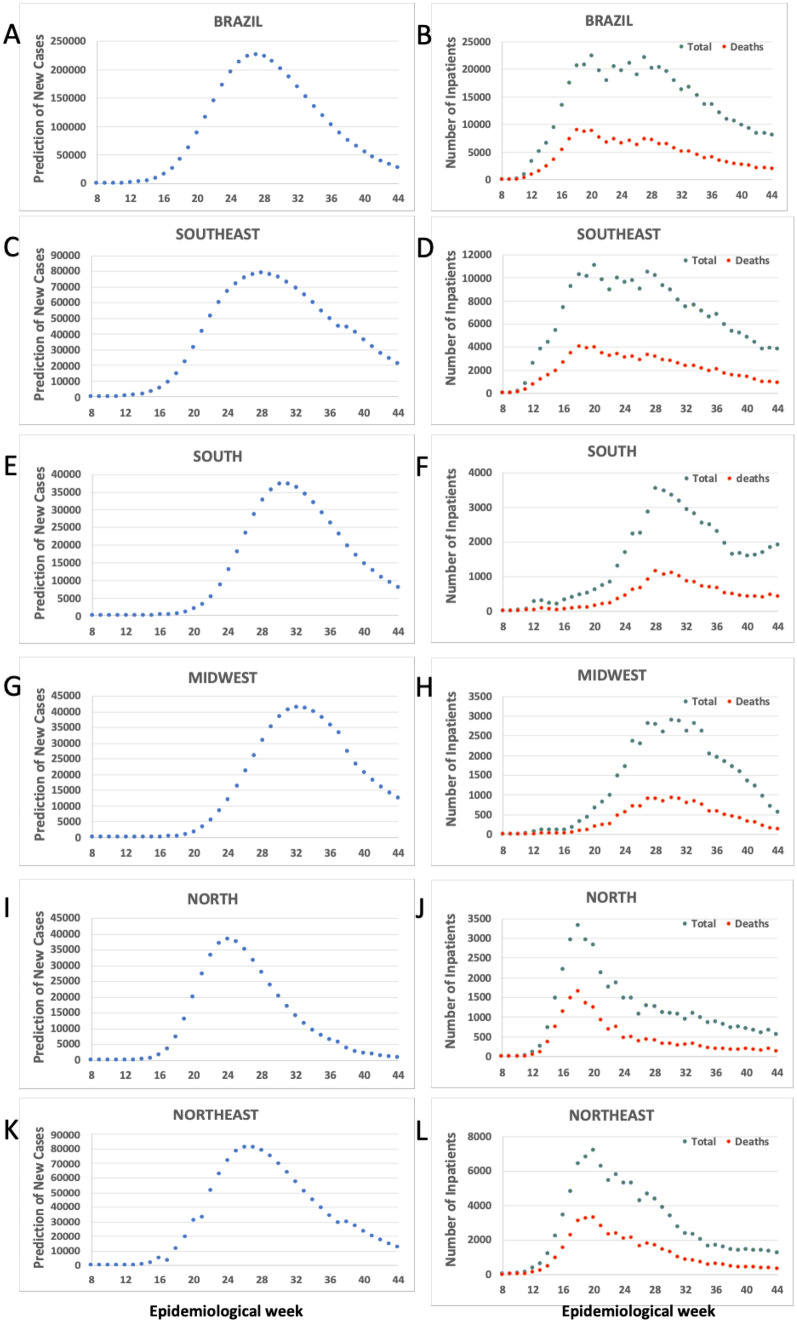
(**A**), (**C**), (**E**), (**G**), (**I**), (**K**): Prediction of new cases of COVID-19; and (**B**), (**D**), (**F**), (**H**), (**J**), (**L**): Total inpatients and deaths related to COVID-19 in Brazil and the five Brazilian regions: Southeast, South, Midwest, North, and Northeast. Analysis was performed from the 8th (Feb 16–22) to the 44th (October 25–31) epidemiological week of 2020.

Brazil and the five Brazilian regions (Southeast, South, Midwest, North, and Northeast) demonstrated that our prediction of new cases of COVID-19 has a good relationship with the total number of inpatients and deaths associated with the disease (Fig. 4). Analysis through the Pearson correlation showed a good positive association; for Brazil, the predicted number of new cases had a correlative value of r = 0.79 (p < 0.001) with the total number of inpatients and of r = 0.66 (p < 0.001) with the number of deaths related to COVID-19. For all the five Brazilian regions, the same strong positive correlation was found: we found a correlation between our predicted number of cases and total inpatients and deaths in the Southeast (r = 0.67 and r = 0.56, respectively; p < 0.001), South (r = 0.96 and r = 0.97, respectively; p < 0.001), Midwest (r = 0.91 and r = 0.89, respectively; p < 0.001), North (r = 0.57 and r = 0.44, respectively; p < 0.01), and Northeast (r = 0.58 and r = 0.48, respectively; p < 0.01).

## Discussion

We widely analyze the general situation of the COVID-19 pandemic in the 26 Brazilian states and their capitals and the Federative Unit. Furthermore, because several interfering and interventional measures, including social distancing/isolating (quarantine and lockdown), mandatory use of face masks, and other government decisions about people lives and economic activities (reopening, flexibilization, and school returning) have been considered fundamental strategies to combat the COVID-19 pandemic^28–30^, we also correlated these measures with the epidemic curve in each state and capital.

There is evidence that susceptibility and mortality related to the COVID-19 pandemic are directly associated with regional differences and preventive/protective measure adoption in various countries^31–34^. Therefore, by evaluating specific Brazilian states and capitals, we could find specific COVID-19 spreading characteristics in a stable period of virus dissemination considering local or regional differences. However, the time for stability of the growth rate of new cases was quite different among the Brazilian states, showing high heterogeneity and complexity of COVID-19 spreading dynamics. Therefore, it is impossible to apply the exact prediction for all Brazilian states without adjusting these discrepancies. Nevertheless, our predictions were performed based on the stable growth rate period and showed an accurate estimation of the COVID-19 epidemic, mainly in a short period (weeks). In addition, because our model is based on logarithmic regression, external factors (e.g., quarantine, mask use, reopening, flexibilization, school returning, etc.) can induce high interference on the epidemic curve.

Our predictions of the cumulative number of cases were accurate in most Brazilian states, mainly in short periods: at day 7, two states of 27 (26 states and one Federative Unit) presented discrepancy higher than 10% between predicted and reported many cases; at day 14, six states; and at day 21, ten states. On day 28, 13 states had differences higher than 10%; in only two states, the discrepancy was higher than 20%. In addition, we could observe that the significant governmental decisions have a great impact on the COVID-19 spread dynamics.

At the end of our prediction time point (September 30, 2020), we observed that the COVID-19 epidemics have specific behavior among the Brazilian states, being classified in three different situations: states with an expected number of cumulative cases close to the predicted number and those with a positive or negative number of cumulative cases (< or > 10%). In addition, we observed that outside factors (preventive measures and governmental decisions about reopening and flexibilization of trading segments) directly interfere with disease epidemics. As a result, the reported number of cumulative cases was higher than the predicted number in the Brazilian states where these extrinsic factors were not completely efficacious to avoid the SARS-CoV-2 spread. On the other hand, when local authorities successfully implemented outside elements to combat the disease epidemic, the reported cumulative number of cases was similar or even lower than the predicted number from our mathematical model.

It is important to highlight that the prolonged duration of the COVID-19 pandemic in Brazil depends not only on the governmental decisions, but also involves several other interfering and inter-related factors that are not yet completely understood yet, including: (a) high economic-social discrepancy with most people living in poor conditions (50% earn less than $ 250 per month and 35% between $250 and $ 600); (b) spatial occupation and isolated communities (homeless, people living in slums, and indigenous communities); (c) insufficient access to public health systems (several Brazilian cities have no intensive care units); (d) misinformation and conflicting decisions among federal, state and municipal governments; and (e) lacking information about age-dependent susceptibility and transmission of the disease (Brazilian National Institute of Geography and Statistics (IBGE), and Brazilian Ministry of Health). One important point to be mentioned is the genetic diversity of the SARS-CoV-2 over the time. Several mutations have been occurred since the virus description, resulting in the emergence of new variants with increased transmissibility^35^, which elevates the virus spreading^36^ and the reinfection risk^37^. The WHO has classified four variants of concern: B.1.1.7 first described in U.K. (Sep 2020), B.1.351 in South Africa (Sep 2020), B.1.1.28.1 in Brazil (Dec 2020), and B.1.617.2 in India (Oct 2020). Because our analysis was performed until September 2020, probably the influence of these variants of concern had no or low impact in this study. However, the influence of other previous variants cannot be discarded.

There are several mathematical models for predicting COVID-19 pandemic evolution since its beginning, most of them based on the classical model SIR^16^. Compared to our model, these models are quite complicated, involve complex computerized calculations and software development, and/or are not completely available or still needing validation, presenting a high discrepancy between the actual data and forecast, even after few days^3^. For instance, SIR model and its derivations depend on various parameters, including those related to epidemiological strategies and virus biology^3^. Usually, these non-trivial parameters are not easy to estimate or calculate, which can result in high discrepancy between the prediction and the real evolution of the pandemic. Therefore, we proposed a straightforward model based on the disease growth rate to predict the Brazilian state's epidemics and capitals. Our model uses easy calculation for the prediction and requires only the cumulative cases of COVID-19 over time, whose data are easily accessible on public websites.

Cotta et al.^38^ used the Susceptible–Infected–Reported–Unreported (SIRU) model to forecast the COVID-19 pandemic in Brazil up to 150 days from February 25, 2020. The model had an excellent prediction in the first 30 days, with less than 10% of difference between the predicted and the reported number of cases. However, after 30 days, the difference was increasing significantly over the time; at 45, 60, and 75 days, the reported number of cases was around 2, 3.5, and 7 times higher than the predicted number, respectively^38^. Using the ARIMA mathematical model, Singh et al.^39^ predicted the total number of COVID-19 cases up to 75 days (from April 24 to July 7, 2020) in several countries. Even in short periods, the difference between the predicted and the reported number of total cases was higher than 20%. Specifically in Brazil, after 30 days, the authors predicted around 175,000 COVID-19 cases at May 24 and the reported cases was about 331,000; at July 7, around 350,000 cases were predicted against about 1,603,000 reported cases (a value ~ 4.5 times higher)^39^. Wang et al.^40^ used a logistic growth forecasting model and machine learning technics to predict the COVID-19 pandemic epidemiology until 200 days in some countries, including Brazil, from June 16, 2020, to January 2, 2021. After 30, 60, 75, and 90 days the reported number was around 25%, 90%, 115%, and 150% higher than the predicted number, respectively^40^. In general, compared with those previous studies, our model was more precise; at 30, 60, 75, and 90 days, the reported number was 9%, 22.3%, 26.7%, and 30.5% higher than the predicted number.

One of the most important aims of the mathematical models is to predict in advance the disease epidemiology and thus to help governmental authorities to establish public health policies to avoid or reduce the overload of health facilities during epidemics. Our model showed a good relationship between the predicted number of new cases and the total number of inpatients and deaths associated with the COVID-19 over the time in the country and its five regions, highlighting the potential relevance of our model in the combat of COVID-19 pandemic. Another importance of the mathematical models is to predict the duration and end of the epidemics. We performed a preliminary analysis about the relationship between the values of alpha or beta factors of our model and the period where the number of new cases start to decrease by 20% or more in the last 14 days. Analysis by the Spearman correlation showed a significant relationship between this period the alpha (r = − 0.801; p < 0.001) and beta (r = 0.862; p < 0.001) factors, suggesting that these factors are potential indicators for forecasting the duration of the COVID-19 pandemic.

Our study has some important points to be highlighted. First, similar to other models^3,39,41^, our prediction accuracy is high in the short-term (weeks), compared to long-term (months). And likewise to other models of logarithmic regression, minor variations in the system, including changes in social distancing and isolating, preventive measures, people mobility, quarantine extension, reopening of non-essential services, stores, and public spaces, as well as other specific and intrinsic factors inherent to each Brazilian state, can lead to significant modifications in the estimated prediction. Thus, by using a stable period of the growth rate to calculate growth and decay factors, we could forecast the number of total cases and evaluate the impact not only of the government interventions but also other interfering factors on disease dissemination.

The second important point is about data accuracy and data time release. There is a delay of some days validating and releasing official data from the Brazilian Ministry of Health and Municipal and State Health Secretaries in Brazil, especially during the weekends. To minimize this variation, we used a 7-days mobile average of the new cases for the analysis.

The third relevant point is that the diagnosed positive cases for COVID-19 are probably lower than the actual number of infected people^42^. In Brazil, the number could be 7–10 times higher than the official reported cases on the Brazilian Ministry of Health. Because most COVID-19 infected people are asymptomatic or present only mild symptoms, the under notification of cases does not devalue our study. Our prediction is mainly for patients who need hospitalization that can overload the public health systems. Various countries have similar criteria for testing COVID-19 suspected people; therefore, our mathematical model can be applied in these countries with similar characteristics to help understand the disease-spreading dynamics.

The fourth important point is that our model needs a period to stabilize the growth rate of new cases, implying that this model cannot apply at the beginning of the pandemic, but only after some weeks with reduced interference of external factors; this is an important limiting factor of our model. This initial stabilizing period is required because of the specific and not completely understood local characteristics and discrepancies among the states. However, in countries with high diversity and complexity of interfering and inter-related factors, like Brazil, where previous studies and predictions did not wholly explain the epidemiology of the COVID-19 pandemic, our model can be handy to understand the behavior of SARS-CoV-2 dissemination in these countries.

## Conclusions

In summary, this is the first study to widely analyze the general situation of the COVID-19 pandemic in Brazil, considering the local/regional characteristics of the states and their capitals and the Federative Unit, using a straightforward mathematical model methodology (GDM). Furthermore, we highlighted the following important points from our work: (1) High heterogeneity and complexity of the regional/local characteristics and governmental authorities among Brazilian states and cities directly influence the COVID-19 spreading dynamics, resulting in different disease epidemic curves; (2) by choosing the best stable period for each Brazilian state and city with reduced or minimal interference on the growth rate of new cases, it is possible to predict the COVID-19 epidemics in the different states and cities with accuracy, mainly in short period (weeks); (3) by plotting the epidemic curves with the main governmental decisions, it is possible to observe the temporal impact of these decisions on pandemic curve growth; (4) a good relationship was found between the predicted number of new cases of COVID-19 by our proposed model and the total number of hospitalizations and deaths related to the disease, highlighting the potential importance of our forecasting in the combat against the COVID-19 pandemic; and (5) our model can easily be applied to follow the evolution of the COVID-19 pandemic, especially in those with persistent pandemic duration, as well as to evaluate the impact of interventional and preventive measures on the disease dissemination, helping and directing health and governmental authorities to make or keep important decisions to combat the pandemic.

## Data Availability

The datasets analyzed during the current study are available in the Brazilian Ministry of Health (https://covid.saude.gov.br/) and World Health Organization (https://covid19.who.int/) repositories.
